# Retrospective Study Regarding the Correlation between Dental Anxiety and Color Preferences in Children with Severe Early Childhood Caries

**DOI:** 10.3390/dj12060155

**Published:** 2024-05-22

**Authors:** Daniela Esian, Cristina Bica, Alexandru Vlasa, Eugen Bud, Elena Stepco, Anamaria Bud, Liana Beresescu

**Affiliations:** 1 Faculty of Dental Medicine, George Emil Palade University of Medicine, Pharmacy, Science, and Technology of Târgu Mures, 540139 Târgu Mures, Romania; daniela.esian@umfst.ro (D.E.); eugen.bud@umfst.ro (E.B.); anamaria.bud@umfst.ro (A.B.); liana.beresescu@umfst.ro (L.B.); 2 “Ion Lupan” Department of Pediatric Oral-Maxillofacial Surgery and Pedodontics, Nicolae Testemitanu State University of Medicine and Pharmacy, 2004 Chișinău, Moldova; elena.stepco@usmf.md

**Keywords:** color preferences, dental anxiety, severe early childhood caries, S-ECC

## Abstract

Background: Severe early childhood caries (S-ECC) is recognized as a significant chronic disease which affects the quality of life starting at very young ages and has a very rapid evolution towards pulp complication and loss of dental tissue. Children with a high caries index DMFT are more likely to develop high levels of dental anxiety, which will influence the degree of cooperation during dental procedures. Emotions play an important role in the psychosomatic development of children, and all the factors that contribute to the modeling of these emotional states must be known and taken into consideration. Aim: The aim of this study was to assess the association between dental anxiety in children with S-ECC and the color preferences for the components of the dental environment to reduce the levels of dental anxiety during medical visits. Material and Method: For this study, 91 children between 3 and 6 years of age diagnosed with S-ECC were selected from the Pediatric Dentistry Department of UMFST from Targu Mures and from a private dental office. The level of dental anxiety was determined by measuring the pulse rate, and a questionnaire was completed to establish the color preferences for the dental office environment and the dentist’s attire. For this purpose, six different colors and their variants with three shades less intensity were chosen by using RGB (Red, Green, and Blue) identification codes for each color. Result: The results showed that there was a statistically significant difference between the age of the subjects and dental anxiety levels, but there was no significant correlation between the DMFT score and dental anxiety. Also, the results showed that there was no significant difference between girls and boys when choosing the colors preferred by them for the dental office, but when comparing the colors that represented happiness, significant statistical difference was found between the girls’ and boys’ groups (*p* = 0.0039). For all the subjects, the colors associated with happiness were light yellow and pink, while the colors associated with anxiety were red and dark blue. Conclusion: The data obtained showed that dental anxiety is strongly influenced by age, and an important role in inducing positive emotions is played by the dental environment if dressed in specific colors in order to reduce dental anxiety and create familiar conditions, especially for young children with S-ECC.

## 1. Introduction

Severe early childhood caries (S-ECC) is a multifactorial disease induced by an imbalance in the tooth hard tissues’ demineralization and remineralization [1], with early onset at very young ages and adverse effects on the child’s esthetic appearance and self-confidence, and with costs for complex treatments. According to the current knowledge, there is a reciprocal relationship between S-ECC and dental anxiety, creating a vicious circle whereby dental anxiety is further sustained by avoiding dental checkups due to the dread of discomfort during dental treatment, which raises the caries index [2].

According to the American Academy of Pediatric Dentistry (AAPD), the definition of ECC is the existence of an extracted tooth or filled tooth surface in the primary dentition of children under the age of 6 due to one or more carious lesions. Every sign of a carious lesion on the enamel of children younger than 3 or a DMFT score higher than age, suggests S-ECC [3].

Dental anxiety, as concerning dental procedures in children, is acknowledged as a concern for public health in numerous nations [4] and could be characterized as a complex reaction made up of the pressure that comes with symptomatic physiologic stimulation, along with an interior sensation of terror [5]. The terms “dental fear” and “dental anxiety” are often used synonymously [6], but the difference between fear and anxiety is the relation between the subject and the stimulus. Dental anxiety represents a condition of understanding that something terrible is about to happen because of dental treatment and is connected to a feeling of losing control, whereas dental fear is defined as a normal emotional response to a particular harmful stimulus. Fear occurs when the stimulus is known and considered dangerous, while anxiety is the reaction developed to an anticipated but unknown stimulus. A serious form of dental anxiety is related to dental phobia, which is manifested by the development of a powerful and constant anxiety regarding the dental environment [7].

The prevalence of dental anxiety ranges from 6% to 20% for kids and teenagers, with an average of 11% [8], and children’s age is a factor with a significant impact on the degree of dental anxiety, with current knowledge claiming that younger kids experience higher levels of anxiety than older kids do when seeing the dentist [9].

The occurrence of dental anxiety is considered to be normal in situations when the possibility of pain is perceived. On the other hand, dental phobia overcomes the barriers of normality through the irrational, intense, and persistent aspect of fear of the dental environment and procedures [10].

Childhood is a period characterized by intense growth and development that take place under the influence of external factors, including a multitude of vibrant colors, and at this age, children create associations between certain colors and different emotional states. Studies from the literature have shown that choosing some child-friendly colors for the dentist’s apparel and the dental office may create a happier environment for the patients with S-ECC and could help to decrease dental anxiety and increase communication between children and the dentist [11].

Goethe introduced a colorful wheel in 1840 that was intended to assess the psychological effects of various hues. It revealed that yellow elicited a happy, upbeat mental state, blue was associated with comfort and calmness, red induced both a positive and negative emotional state, orange was associated with stress, green induced fear, and black was the color associated with anxiety and depression [12].

Several indirect techniques, such as tracking physiological reactions like sweating and heart rate and inspecting children’s attitude during dental visits, can be used to evaluate children’s dental fear and anxiety. Since pulse rate is thought to be a physiological predictor of dental anxiety, pulse oximeters have been recognized as a trustworthy tool for measuring it directly in dental settings. A high level of dental anxiety and stress during a dental visit is correlated with an elevated pulse rate [13].

The present research aimed to analyze possible correlations between different levels of dental anxiety in children with S-ECC.

## 2. Materials and Methods

The study was conducted between June 2023 and October 2023 in the Pediatric Dentistry Department of UMFST G.E. Palade of Targu Mures and in a private dental office according with the Declaration of Helsinki and approved by the Ethics Committee of University of Medicine, Pharmacy, Science and Technology “G.E. Palade” of Targu Mures (No. 2169/09.03.2023). Before beginning the survey, the parents of all children were asked for written agreement, and the subjects were given information about the study’s protocol and goal. A sample size calculator (Qualtrics™, version 1, Seattle, WA, USA, for Windows™ Excel, version 1, Redmond, WA, USA) was utilized to ascertain whether the study’s sample size was sufficient to allow for conclusions about the correlation between dental anxiety and color preferences in children. Sample size was determined based on the literature [14,15] by using the power analysis calculation, which was set to provide a standard error of 5% and 95% confidence interval. The defined prevalence of DA in children aged 3–6 was 28.9% was, and 100 was the computed sample size. The ideal sample size would have been achieved if the initial number of individuals had included nine additional patients. We see this as a weakness in our research and hope to increase the sample size in the future to get more measurable results. Given that other researchers have observed comparable results with larger subject groups, the current results provide a solid basis for further research on this topic and cannot be disregarded [14,15]. The sample of the subjects consisted of 91 children diagnosed with S-ECC who were between three and six years of age at the time of data collection. Based on age, the subjects were split into two groups, respectively, a group aged between 3 and 4 years and a group aged between 5 and 6 years. According to gender, the subjects were divided into a group of girls (*n* = 45) and a group of boys (*n* = 45).

The following factors were taken into consideration when choosing the study’s subjects:

Inclusion criteria: children aged 3–6 years with S-ECC who attended the dental office for checkup and current treatment, children with adequate psychological and mental condition.

The number of primary teeth that were found to be decayed, filled, or missing after a clinical examination in accordance with the World Health Organization (WHO) 1997 criteria was added together to determine the DMFT index. Missing teeth were those that had been extracted because of dental caries.

In accordance with AAPD [3], the primary S-ECC diagnostic criterion for participants in the study group was a DMFS score of greater than or equal to age, or a DMFT score of higher than or equal to four (age 3), higher than or equal to five (age 4), higher than or equal to six (age 5), and higher than or equal to seven (age 6).

Exclusion criteria: children out of age group, children with caries other than S-ECC, children diagnosed with color blindness, and children with a low degree of cooperation.

Color blindness was excluded based on the medical history taken in the presence of the parents.

### 2.1. Study Design

The present retrospective research pursued two main objectives to assess the degree of dental anxiety related to age of the subjects by using pulse oximetry to measure the pulse rate and to determine the correlation between color preferences related to the design of the dental environment and certain emotional states by completing a questionnaire.

Under operating light, oral examinations were conducted in a dental unit, and the total DMFT score was computed. The individuals’ ages and dental anxiety levels were correlated with the caries index DMFT.

By taking their heart rates at various stages during the dental visit, the subjects’ level of dental anxiety was ascertained: the first momentum was when patients remained in the waiting room, and the second was determined immediately after the dental appointment.

A fingertip pulse oximeter (YK-81C Silver) was used to capture the pulse rate. The pulse rate was measured two times, and the mean pulse rate was calculated. Data were collected and analyzed by software specifically designed for digital processing.

Since heart rate is the simplest biological parameter to measure and has been validated by other researchers [16], it was selected for the assessment of dental anxiety because, as prior studies have shown, it is more consistent with anxiety episodes during dental visits than other physiological parameters [17].

Later in the study, a 10-item self-completion survey was created and utilized to gather general information about the dental visit and also the subjects’ color preferences related to the design of the dental environment, like dental office (walls and dental chair) and the dentist’s attire (scrub and mask), with each of these items being presented in the form of a drawing in 12 different shades and colors.

For this purpose, 6 different colors and their variants with 3 shades less intensity were chosen, and the website plainblackground.com was used to select the color shades. The following table shows the RGB (Red, Green, and Blue) identification codes for each color (Table 1).

### 2.2. Statistical Data Analysis

Using Microsoft Excel™ work sheets (Microsoft Corporation, Washington, DC, USA, version 2018), data regarding the findings of the conducted research were collected. For the evaluation of the statistical analysis, GraphPad Prism version 8.0.0 for Windows (GraphPad Software, San Diego, CA, USA) was used. Descriptive statistics such as mean, standard deviation, median, minimum, and maximum value, for each group of data were assessed. Using the Kolmogorov–Smirnov test, the data normality was calculated. The differences regarding the color preference between the two groups was determined by applying the Wilcoxon and Friedman tests. The chosen significance level was set at 0.05.

## 3. Results

The total number of the subjects included in this study was 91, of which 49.45% were girls (*n* = 45) and 50.55% boys (*n* = 46). The distribution of children aged between 3 and 6 years was as follows: 3 years, 14%; 4 years, 21%; 5 years, 30%; and 6 years, 35%, in approximately equal proportion according to gender (Table 2).

**Figure 1:**
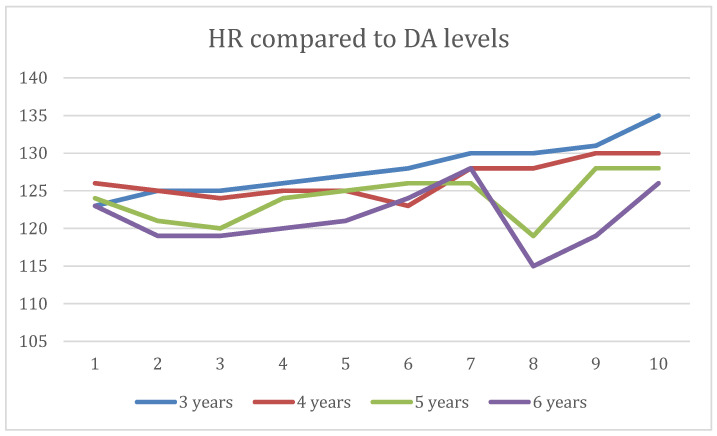
Correlation between DA and pulse rate in the study groups according to age.

When comparing the degree of DA among age groups, it was shown that the child’s age and dental anxiety ratings were highly correlated (*p* < 0.05), so there was a tendency for the DA level to decrease with age. The comparison between the frequency of the DA levels at all ages was not statistically significant (*p* = 0.1371). There was no significant difference between close age groups, respectively, between 5 and 6 years of age (*p* = 0.5092), but there was a significant difference regarding DA levels between the 3- and 5-year-old age groups (*p* = 0.0273) (Figure 1).

**Figure 2:**
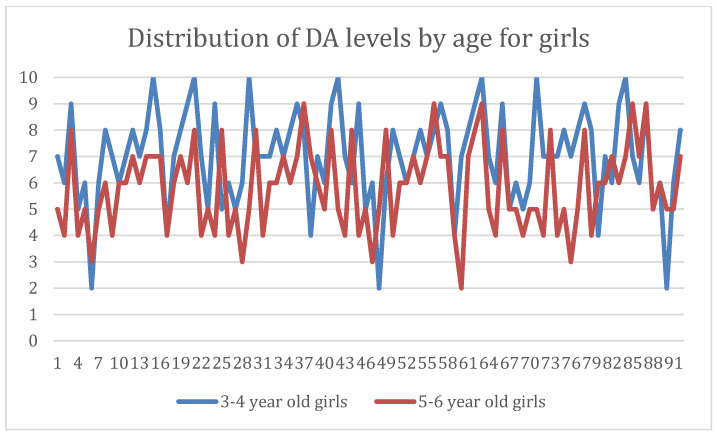
Distribution of dental anxiety in girls within the study groups.

**Figure 3:**
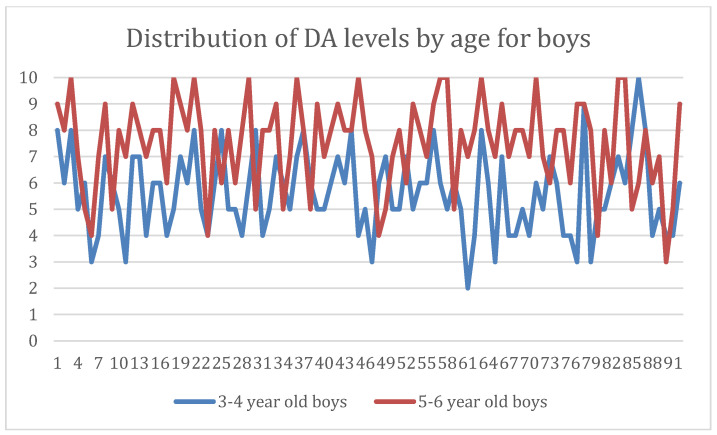
Distribution of dental anxiety in boys within the study groups.

When dental anxiety levels were compared by gender, the findings indicated that, in contrast to the boy’s group, where anxiety levels rise in direct proportion to age, girls in the first study group, who were between the ages of 3 and 4 years, had higher levels of dental anxiety. In the second study group, consisting of girls between 5 and 6 years old, however, we observed lower DA levels (Figure 2 and Figure 3).

The total mean of the DMFT score was 7.79 (SD 4.7), the mean of DMFT was 7.64 (SD 5.1) for girls and 8.07 (SD 5.2) for boys. No significant correlation was found between dental anxiety levels and DMFT index values (*p* > 0.05).

When comparing the color preferences that represented happiness between the girls’ and boys’ groups, a significant difference was found (*p* = 0.0039). The comparison between girls and boys when choosing the colors that were associated with anxiety did not show significant differences (*p* = 0.8457). When comparing all subjects’ color preferences associated with happiness vs. anxiety, the difference was not statistically significant (*p* = 0.1763) (Table 3 and Table 4).

No statistically significant difference was found between girls and boys when choosing the color preferred by them for the dentist’s scrub (*p* = 0.6953)*,* the dental chair (*p* = 0.5703)*,* the dentist’s mask (*p* = 0.4648), or the dental office’s walls (*p* = 0.76954). Also, there was no statistically significant difference between the subjects’ color preferences for the scrubs, dental chair, mask, and the office walls (*p* = 0.9823) (Table 5 and Table 6).

For the 3-year-old subjects, there was a clear tendency to pick color number 6 (*Persian Pink*) for the dentist’s scrub, mask, dental chair, and dental office walls, while for the 4-year-old subject group, the color preference was variable, with a slight tendency towards color number 5 (*Light Persian Pink*). Regarding the 5-year-old subjects, the color preferences revolved around color numbers 10 and 11 (*Chinese Red and Light Porcelain Blue*), while in the 6-year-old subject group, the tendency was again towards color number 5. The colors associated with happiness were light yellow and pink for all the subjects, while the ones associated with anxiety were red and dark blue.

## 4. Discussion

The present clinical trial aimed to correlate the level of dental anxiety in patients with S-ECC according to age and to identify the color preferences in reducing dental anxiety. A total of 91 subjects, aged between three and six years, with S-ECC were selected to determine the level of dental anxiety based on their pulse rate, and a questionnaire about the chromatic preferences of the dental office environment and the dentist’s attire was completed.

An increased likelihood of developing carious lesions in both the primary and permanent dentition, hospitalization, and ER visits, missed school days, and a lower quality of life related to oral health are all common outcomes of S-ECC [1]. Children’s dread and anxiety can be heightened by an unpleasant experience, and inadequate dental management is closely linked to dental anxiety in young patients [1].

In order to prevent dental anxiety in children with S-ECC, minimally invasive techniques are recommended in association with glass-ionomer preventive fillings. If complex restorations are needed, the Hall technique can be used as a minimally invasive treatment by applying a stainless-steel crown directly over the carious lesion. It is important to monitor the restoration to the physiological eruption of the first permanent tooth and the extraction of the primary tooth if resin-modified glass-ionomer cements are used [18,19,20].

Regarding the degree of dental anxiety, according to gender, our results revealed a higher level of dental anxiety in girls in the first age group of 3–4 years compared to the second age of group. With increasing age and the transition to mixed dentition, the girls/boys’ ratio reverses, with the higher level of dental anxiety being found in the boys’ group of subjects.

Data from the literature suggested that girls are more likely to develop dental anxiety than boys, who tend to hide their fear [21].

Other studies indicate higher values of dental anxiety observed in subjects of younger age, especially in girls [22,23].

Most studies reported higher levels of dental anxiety in girls, with this aspect also being correlated with ethnic and economic status and poor education. Thus, it was observed in the cultural and ethnic context that some communities inhibit the expression of emotions, with children presenting an unexpected reaction of apparent calm even in conditions of mental stress. In this context, a vicious circle is created: dental anxiety prevents dental procedures from being carried out, and late presentation in advanced stages of S-ECC increases the level of dental anxiety. It was also observed that the increased number of siblings directly and proportionally influences the level of dental anxiety [8,22,23].

Regarding the level of dental anxiety related to the age groups, a tendency to decrease with age was observed in our study, with subjects in the age group of 3–4 years being more anxious than subjects from the age group of 5–6 years. Similar to these results, prior research has demonstrated that smaller kids are more afraid of the dentist than older kids when they visit the dental office [24]. The most likely cause of this is that younger children often experience feelings of uncertainty and abandonment. Compared to younger children, those above the age of five have superior comprehension and reporting skills for all areas of their health, including their emotional state [25]. These could be caused also by a careless perspective on the importance of maintaining the health of primary teeth, but there are also some social factors, such as socioeconomic factors, the inability to access dental services due to financial considerations, and or the education level of the parents.

In the realm of color, modern marketing strategies are thought to be largely responsible for the two parallel gender images that have been created: girls’ pink and boys’ blue. The clothes that the little ones wear and the toys that surround them are chosen by the parents, usually according to the gender. Therefore, from an early age, children begin to perceive certain colors as gender-related, often ending up rejecting others on the ground that they do not match the gender with which the child identifies [26].

As concerning the chromatic preferences, the results of the present study support this hypothesis because, if in younger subjects there is a diversity of chromatic preferences in the subjects of the last group of age (6-year-old), more grouped responses are observed according to the model: girls = pink, and boys = blue/green.

The initial clinical communication channel between the patient and the dentist is the dentist’s outfit. Consequently, color preference may be assessed to ascertain its impact on pediatric patients’ anxiety levels and their contentment with the dental setting [27].

However, vibrant colors activate the senses and release adrenaline and cortisol, which raise blood pressure, especially in tiny patients, by inducing tension and anxiety [28].

In a study designed by Annamary et al. [15], it was demonstrated that, in a dental clinic, colors like blue and pink help boost a positive attitude and lessen dental fear, whereas red and dark hues accentuate a negative attitude.

In the present study, in girls from all study groups, we observed a preference for pink and yellow colors, including both the less intense and the more pigmented shades. Boys seemed to show a greater receptivity to the chromatic background, and a more pronounced variability of the responses was observed. For this reason, most evaluations that take as reference the entire group of subjects seem to reveal the choices of girls where a solidarity towards a limited number of options is highlighted.

Evaluating the results according to gender, we see that there is a tendency of girls to choose the pink color, while they declare that green and blue cause them anxiety. On the other hand, boys seem to prefer precisely green and blue, accusing the red-orange tones of inducing states of anxiety.

A Corah dental anxiety measure was used in a study by Bubna et al. [29] to assess children aged from 6 to 12, and the results showed no discernible variations in the subjects’ color preferences between the two groups, namely those with and without dental anxiety. As a result, both groups were very interested in choosing yellow to represent happiness, while both groups’ participants chose red to represent discomfort.

Due to the fact that the color yellow is associated with happiness in all age groups in a significant proportion in the present study and also in other studies [30,31,32,33], and because it is a gender-neutral color, not being strongly correlated with the image of femineity or masculinity, this color could form the core of dental office design and dentists’ attire.

An important role in reducing dental anxiety in children with S-ECC is played by both the parents and the dentist. Thus, the parents must establish early and frequent visits to the dentist in the absence of a specific emergency treatment so that the child becomes familiar with the dental environment without additional stress, and the dentist can contribute to reducing anxiety by creating a warm environment dressed in cheerful colors.

Our study’s main limitation consists of the small number of children we examined and if the results would have been more accurate if nine more patients would have been included in the initial number of subjects, as the sample size would then have been optimal.

Another limitation refers to color blindness as a possible undiagnosed condition, particularly in younger children, if we take into account the medical history as the only method to exclude this condition.

Considering these limitations, additional clinical trials on a larger group of subjects are required to assess the implication of a more accurate diagnosis of color blindness and the possible advantages of this strategy.

## 5. Conclusions

Children with S-ECC are more likely to develop dental anxiety, due in part to early onset at very young ages and in part to the discomfort and pain that follow the rapid progression of caries to extensive complications.

This study highlights a tendency for the level of dental anxiety to decrease with age in girls, while in boys, the ratio reverses, and higher levels of dental anxiety are observed after the age of 4–5 years old.

The color preferences of boys and girls regarding the colors associated with happiness and fear are different, but there is a prevalence for all subjects to associate happiness with light yellow and fear or anxiety with red and dark blue.

## Figures and Tables

**Table 1:** Identification codes for colors RGB.

Color Number	The Name of the Color	RGB Code
1	Light Deep Green	(79, 148, 81)
2	Deep Green	(0, 102, 2)
3	Light Philippines Orange	(255, 158, 75)
4	Philippines Orange	(255, 116, 0)
5	Light Persian Pink	(249, 167, 210)
6	Persian Pink	(247, 128, 191)
7	Light Absolute Yellow	(255, 224, 82)
8	Absolute Yellow	(255, 211, 2)
9	Light Chinese Red	(211, 80, 97)
10	Chinese Red	(206, 1, 24)
11	Light Porcelain Blue	(97, 134, 176)
12	Porcelain Blue	(24, 81, 141)

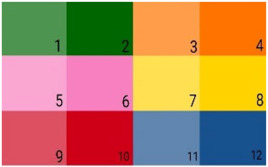

**Table 2:** Distribution of subjects according to age groups and gender.

Age Group	Number of Subjects	Girls	Boys	Total
3–4 years of age	N (%)	18 (56.25)	14 (43.75)	32 (35)
5–6 years of age	N (%)	27 (47.76)	32 (54.23)	59 (65)

**Table 3:** Distribution of color preferences at 3 years old, according to gender.

	3-Year-Old Subjects		Girls		Boys	
	Color	%	Color	%	Color	%
Happiness	6	27	6	37	7	27
Anxiety	12	27	12	37	10, 11	18
Dentist’s scrubs	6	23	6	37	12	27
Dental chair	6	37	6	58	4	36
Mask	6	30	6	42	3	18
Walls	5	27	5	32	5, 7	18
Medical cap	6	27	6	37	3, 4	18

**Table 4:** Distribution of color preferences at 4 years old, according to gender.

	4-Year-Old Subjects		Girls		Boys	
	Color	%	Color	%	Color	%
Happiness	7	29	7	27	7	30
Anxiety	12	33	12	55	9, 10	30
Dentist’s scrubs	5	29	5	45	2	30
Dental chair	6	23	5	55	12	30
Mask	2	23	5, 6	27	2	50
Walls	7	38	5	45	7	50
Medical cap	5	29	5	45	2	50

**Table 5:** Distribution of color preferences at 5 years old, according to gender.

	5-Year-Old Subjects		Girls		Boys
	Color	%		Color	%
Happiness	7	36	-	7	45
Anxiety	10	43	-	10	45
Dentist’s scrubs	10	29	-	10	36
Dental chair	10, 11	21	-	10, 11	27
Mask	10, 11	21	-	10, 11	27
Walls	7	43	-	7	45
Medical cap	11	29	-	11	36

**Table 6:** Distribution of color preferences at 6 years old, according to gender.

	6-Year-Old Subjects		Girls		Boys	
	Color	%	Color	%	Color	%
Happiness	6	31	6	53	10	28
Anxiety	12	17	2	29	3, 4, 10, 12	17
Dentist’s scrubs	5	23	5	35	1, 10	33
Dental chair	5, 11	23	5	35	11	44
Mask	5	29	5	47	11, 12	22
Walls	5	29	5	35	11	22
Medical cap	5	17	5	24	1	28

## Data Availability

All data regarding this manuscript can be checked with the corresponding authors.
